# Influenza Vaccine Coverage among Pregnant Women in a Public Hospital System during the 2009-2010 Pandemic Influenza Season

**DOI:** 10.1155/2012/329506

**Published:** 2012-05-07

**Authors:** Dean V. Coonrod, Blanca-Flor Jimenez, Amber N. Sturgeon, David Drachman

**Affiliations:** ^1^Department of Obstetrics/Gynecology and Women's Health, Maricopa Integrated Health System, Phoenix, AZ 85008, USA; ^2^Department of Research, Maricopa Integrated Health System, Phoenix, AZ 85008, USA; ^3^Department of Obstetrics and Gynecology, District Medical Group, Phoenix, AZ 85016, USA; ^4^Department of Obstetrics and Gynecology, University of Arizona College of Medicine, Phoenix, AZ 85008, USA

## Abstract

The purpose of this study was to compare influenza vaccination rates of pregnant women in a public safety-net health system to national coverage rates during the 2009-2010 pandemic influenza season. A chart review of a random sample of deliveries was undertaken to determine rates of coverage and predictors of vaccine coverage of women who obtained prenatal care and delivered in our health system. Rates were calculated from deliveries from when the vaccine was first available through April 30, 2010. Coverage rates were 54% for the seasonal influenza vaccine and 51% for the H1N1 vaccine. Race/ethnicity, insurance status and language spoken did not predict the receipt of either vaccine. When we included only births which occurred through March 12, 2010, as was done in a large population-based study, the rates were 61% and 59%, respectively. Our rates are about 10% higher than the rates reported in that study. Our comprehensive strategy for promoting vaccine coverage achieved higher vaccination rates in a safety-net health system, which serves groups historically less likely to be vaccinated, than those reported for the pregnant population at large.

## 1. Introduction

Currently the influenza vaccine is recommended in the United States for all individuals over 6 months of age [1] and by the World Health Organization for high-risk groups [2]. These recommendations have, in part, the aim to reduce the worldwide estimated deaths 250,000–500,000 each year caused by the seasonal form of the disease [3]. Pregnant women are included in these recommendations because vaccination with the inactivated virus has been shown to decrease the burden of suffering among neonates [4–6] and to reduce maternal morbidity of the infection [7]. Furthermore, vaccination in pregnancy has been documented as sufficiently safe to be recommended to be given regardless of trimester [4, 8].

 Despite these recommendations in pregnancy, coverage of pregnant women in the United States has been low in the years prior to the 2009-2010 influenza season (11% to 24%) [7]. Reasons cited for this include not being offered the vaccine, not having the vaccine in the office, and maternal concerns for vaccine safety [9, 10].

In the spring of 2009, a new swine-origin form of the influenza A virus, causing substantial morbidity and mortality, was isolated: H1N1 [11]. As part of the pandemic strategy for this virus, a specific monovalent vaccine for H1N1 was developed and was ready for distribution in the Fall of 2009 [12]. Among the high-risk groups targeted to receive this vaccine, in addition to the seasonal vaccine, were pregnant women [12]. The rationale for this included the high mortality rates associated with influenza among pregnant women in earlier pandemics [13] and early reports of the severity of H1N1 illness in pregnancy, [14–16] which proved to be true as the 2009 to 2010 influenza season progressed [17, 18]. Recently, population-based data on vaccine coverage of pregnant women in the United States during the 2009-2010 pandemic were published by the Centers for Disease Control (CDC) [19]. This report revealed less than full coverage during that season despite a robust public-health response, with self-reported coverage rates of 51% for the seasonal vaccine and 47% for the H1N1 vaccine. The purpose of this study is to compare these national rates to those in a public safety-net hospital system serving a large number of uninsured patients, using chart-verified documentation of vaccination.

## 2. Methods

This was a retrospective cohort study of pregnant patients who delivered at Maricopa Medical Center during the 2009-2010 influenza pandemic. In order to be eligible, subjects must have received prenatal care at the hospital or one of the nine associated prenatal clinics preceding their delivery. Maricopa Integrated Health System (MIHS) includes Maricopa Medical Center and associated clinics. It serves as a public safety-net facility for Maricopa County (population 3.8 million) which includes Phoenix, Arizona. Efforts to promote influenza vaccine coverage for pregnant patients during the 2009-2010 influenza season included education of providers via grand rounds, small group in-service sessions, and emailed recommendations for clinicians providing prenatal and intrapartum care. Standing orders for nursing staff to administer the vaccine without a physician signature were also written and distributed. Indirect efforts to promote vaccination included activities to increase awareness, such as signage regarding influenza, distribution and implementation of influenza-like illness (ILI) protocols, phone followup of ILI patients not requiring admission, and backup call schedules for attending physicians, as well as infection control activities with screening of patients and visitors for ILI in our health care facilities. We did not have an electronic medical record with a vaccine registry to identify prospectively those who had not been previously vaccinated.

In order to be consistent with the CDC's study [19] we included births which occurred from when the vaccines were first available in our health system, September 15, 2009 for the seasonal vaccine and October 15, 2009 for the H1N1 vaccine. A random sample of subjects was selected for review from a billing database of 939 deliveries which occurred during the September 15, 2009 to April 30, 2010 time frame. Charts were first reviewed for eligibility: receipt of prenatal care at MIHS. At the beginning of the chart review, a sample of charts (the first 10 charts by each reviewer) was reviewed by a second reviewer to assure accurate capture of the study variables. In addition, in order to be certain that vaccine administration was not missed in the chart review, a second review of charts with no documented vaccination was undertaken by a different reviewer. Predictor variables were also collected during the chart review and included age at delivery, gestational age at delivery, race/ethnicity, patient language, and gravidity and parity. The billing database served as the source of information for insurance status. The outcome was receipt of the seasonal vaccine and/or the H1N1 vaccine as determined by documentation on progress notes or vaccination forms.

Our targeted sample size was 200, which would allow us to be at least 95% certain that the observed vaccination rate falls within 6.9% of the true value. Prevalence rates of vaccine coverage and 95% confidence intervals were calculated for the 2 vaccines for different time periods as noted in Section 3. Similarly, prevalence of vaccine coverage was calculated for subcategories of the predictor variables. Chi-square statistics were used to determine statistical significance with a *P* value of <0.05 considered significant. This study was approved by the MIHS Institutional Review Board via expedited review on November 10, 2011.

## 3. Results 

 A random sample of 296 of the 939 deliveries which occurred in the study time frame was reviewed. Of these, 50 were ineligible because of no prenatal care at MIHS. The seasonal influenza sample comprised a total of 246 women who delivered between September 15, 2009 and April 30, 2010, and the H1N1 sample comprised. 217 who delivered between October 15, 2009 and April 30, 2010 Table 1 provides a description of the seasonal influenza sample. The sample was representative of our population; mainly Hispanic, non-English speaking, without insurance, and of high parity.

 In the seasonal influenza sample, 132 of the 246 subjects (54%) received the seasonal vaccine, (95% confidence interval, 48% to 60%). In order to more directly compare the rates with national rates as presented by the CDC [19], the sample was restricted to births occurring until March 12, 2010. Of these 216 subjects, 61% received the vaccine (95% confidence interval, 55% to 68%).

 In the H1N1 sample, 111 of 217 (51%) received the H1N1 vaccine, (95% confidence interval, 44% to 58%). In the sample restricted to births through March 12, 2010, 111 of 187 (59%) received the H1N1 vaccine (95% confidence interval, 52% to 66%).

 The corresponding rate for receipt of both vaccines from October 15, 2009 to April 30, 2010 was 33% (72 of 217) (95% confidence interval, 27% to 40%). When restricted to births through March 12, 2010, the corresponding rate was 39% (72 of 187; 95% confidence interval, 32% to 46%).

 Our commitment to vaccinate against influenza required extra, nonroutine efforts in the form of education and mandatory separate written orders or “standing orders,” both of which involved separate paperwork. To understand the role of the additional documentation effort, we compared rates of influenza vaccination with rates of vaccination associated with the tetanus, diphtheria, and pertussis (Tdap) vaccine that is written into all preprinted postpartum orders. A total of 201 of 246 (82%) received the Tdap vaccine (95% confidence interval: 76% to 86%).

 Predictors of vaccination were also examined (Table 2). None of the demographic and reproductive characteristics were significantly related to the receipt of either the seasonal or the H1N1 vaccine. This indicates that, in our health system, primary language, race/ethnicity, and even insurance status were not barriers to the receipt of the vaccine. The only significant predictor in Table 2 was receipt of the other influenza vaccinse, that is, receipt of the seasonal vaccine was predictive of a higher likelihood of receiving the H1N1 vaccine and vice versa.

## 4. Discussion

 In our health system during the 2009-2010 influenza season, we found rates of vaccination of pregnant women of 54% for the seasonal influenza vaccine and 51% for the H1N1 vaccine. When restricted in a manner similar to a 10-state sample [19] (births through March 12, 2010) our rates were approximately 10% higher: seasonal vaccine coverage was 61% versus 51% and H1N1 coverage was 59% versus 47%. This is notable since our population serves pregnant women who have been shown in previous studies to be less likely to receive the vaccine, those who are disadvantaged, that is, uninsured, nonwhite, less educated, and of low income [20–22].

 Other studies have reported on vaccination coverage of pregnant women in the 2009-2010 pandemic influenza season, many of which rely on self-report. In the United States, a nationally representative phone survey found coverage rates of 32% for the seasonal vaccine and 46% for the H1N1 vaccine [23]. Another nationally representative phone survey found similar results, 42% for the H1N1 vaccine [24]. Higher rates have been reported from samples in health care institutions. A study at a university hospital in Denver, CO, found higher coverage rates of 64% for the seasonal vaccine and 54% for the H1N1 vaccine [25]. Unlike our study, the Denver study relied on self-report and demonstrated high rates of vaccination outside their health system, 15% for the seasonal vaccine and 29% for the H1N1 vaccine. We do not know the magnitude of the outside vaccination in our population. A high rate of H1N1 vaccine coverage (76%) was achieved in a public hospital prenatal clinic in Seattle, WA, using a system which included chart prompts and a vaccine registry [26]. The highest rates were reported at the Massachusetts General Hospital with coverage rates of 88% and 86% for the seasonal and the H1N1 vaccines, respectively [27]. Their sample was restricted to 3 months of the influenza season (January 2010 to March 2010) which could have raised these estimates as compared to other studies, since both vaccines were more readily available at that time. A time difference might in part explain the lower coverage rates of the H1N1 vaccine (26%) reported in one health system, Maimonides Medical Center in Brooklyn, NY, as the sample began shortly after the H1N1 vaccine was available (time frame of November 12 to December 19, 2009) [28]. Coverage rates outside the United States have also been reported. In Canada, rates of seasonal coverage were reported to be 30% in Alberta [29], while for H1N1 the rates have been reported to be between 63% and 76% [29, 30]. In France, rates of the H1N1 coverage of pregnant women were lower: 38% in a 3-hospital study in Paris [31] and 22% using a national insurance database [32]. Another insurance database from Australia reported even lower estimated coverage rates of 10% for the H1N1 vaccine in pregnant women from October 2009 to January 2010 [33].

With one exception, all of the rates are lower than the 80% coverage rates targeted by the national Healthy People 2020 objectives for pregnant women [34]. However, most represent large increases over the prior year rates [7], which appear to be sustained with 49% pregnant women reporting being vaccinated during the 2010-2011 influenza season [20]. In our health system we achieved rates higher than nationally reported rates; we attribute this to a comprehensive approach to influenza. For example, we undertook many of the prevention strategies cited by Goldfarb et al. [27] in their successful program. Our actions included standing orders [35] and awareness activities, [36] both of which have been demonstrated to increase vaccination rates. Reasons for our rates being lower than the highest reported rates may have included not having chart prompts [37] or not making use of a vaccine registry [26]. Furthermore, our system of prenatal care includes clinics spread over a large geographic area, making messaging and implementing programs challenging. However, streamlining the order process as we have been able to do with the Tdap vaccine suggests that even higher rates can be achieved.

Our study has a number of strengths. It was based on chart review which indicates that we can be relatively certain of the lower limit of our vaccination rates. It is possible that actual coverage rates may be higher, especially in view of the high rates of vaccination outside of the health system reported by Fisher et al. [25]. However, not knowing the magnitude of the outside coverage in our population, we would not want to speculate on how much higher the “real” coverage rates would have been. Another advantage of the chart review methodology is that many of the studies rely on self-report, which may have been an issue in the 2009 to 2010 season when 2 vaccines were available. Our own experience with our population suggests this may have occurred, with a frequent response of, “I already received the flu vaccine,” when they had received only one of the recommended two vaccines. Furthermore, many of the studies relying on self-report had substantial nonresponse rates of approximately 50%; this included studies with high reported coverage rates or those that were nationally representative [23, 25, 27]. A limitation of our study is that it represents the cumulative efforts of one health system caring for the underserved, as such these results may not be generalizable to other populations or health systems. Finally, we do not know which of our interventions impacted vaccination rates the most.

 Our study has a number of implications. For other health systems it would appear that improved vaccination rates as compared to the national rates are possible, even for health systems that are safety-net systems that provide medical care to large numbers of underserved patients. Our success with a population which usually experiences lower coverage rates—minorities, uninsured, and with limited English-speaking ability—could be a model for other systems. Taken with Goldfarb's study [27], we also believe a comprehensive approach might be a rational strategy to achieve high coverage rates. Given initial reports of continued high rates in pregnant patients over the subsequent influenza season to this study, it will be interesting to see if high vaccination rates continue in this population. Finally, given that self-report might overestimate and chart review might underestimate coverage rates, it would be interesting to supplement chart review with self-report to further characterize this in our population. Moreover, self-report in such a study would assist in determining the upper limit achievable by segregating those who represent missed opportunities versus those who refuse the vaccine.

 In summary, our public safety-net institution achieved influenza vaccine coverage rates, that were higher than those reported national samples by instituting a comprehensive strategy. Our rates however, were not as high as those in reported in “best practice” studies. This could be due to not capturing vaccinations done outside our health system, not including certain interventions in our vaccination strategy such as chart prompts, and not including an order for influenza vaccination on all postpartum orders.

## Figures and Tables

**Table 1:** Demographic and reproductive characteristics of the study sample of 246 deliveries from the 2009-2010 influenza season (delivery dates September 15, 2009, to April 30, 2010).

Characteristic	*N*	%
Age*		
<25	84	34%
25–35	132	54%
>35	29	12%
Race		
White non-Hispanic	11	4%
Hispanic	209	85%
African American	11	4%
Asian	2	1%
Other/unknown	13	5%
Language*		
Any English	75	31%
Spanish only	167	69%
Insurance^†^		
Uninsured	207	84%
Insured	39	16%
Parity		
0	49	20%
1	59	24%
2+	138	56%
Gestational age*		
<37 weeks	19	8%
37+ weeks	220	92%

*Totals may sum to less than 246 due to missing values. ^†^Indicates insurance status for patient's prenatal care.

**Table 2:** Numbers and percentage of patients vaccinated by demographic and reproductive characteristics.

Characteristic	Seasonal vaccine		H1N1 vaccine	
	*N* = 246		*N* = 217*	
	*n*/*N* ^†^	%	*n*/*N* ^†^	%
Age				
<25	50/84	60%	35/69	51%
25–35	66/132	50%	60/121	50%
>35	15/29	52%	13/27	48%
Race				
White non-Hispanic	4/11	36%	4/10	40%
Hispanic	112/209	54%	92/183	50%
African American	7/11	64%	5/11	45%
Language				
Any English	40/75	53%	33/66	50%
Spanish only	90/167	54%	73/147	50%
Insurance^‡^				
Uninsured	115/207	56%	92/182	51%
Insured	17/39	44%	16/35	46%
Parity				
0	29/49	59%	24/41	59%
1	32/59	54%	22/53	42%
2+	71/138	51%	62/123	50%
Gestational age				
<37 weeks	8/19	42%	4/15	27%
37+ weeks	120/220	55%	103/197	52%
Receipt of the other influenza vaccine^§^				
Yes	72/111	65%	69/115	60%
No	60/135	44%	39/102	38%

*Sample drawn from deliveries from October 15, 2009, through April 30, 2010 (after the H1N1 vaccine first became available). *n* ^†^: number vaccinated; *N*: number in subgroup. ^ ‡^Indicates insurance status for patient's prenatal care. ^§^ *P* < 0.05 for both types of vaccines.
